# Bilateral tonsillar infiltration of T‐cell prolymphocytic leukemia

**DOI:** 10.1002/ccr3.2418

**Published:** 2019-09-18

**Authors:** Ilaria Bertaggia, Sabrina De Stefano, Filippo Di Lella, Giovanni Roti

**Affiliations:** ^1^ Department of Medicine and Surgery University of Parma Parma Italy

**Keywords:** acute medicine, hematology

## Abstract

Tonsillar lymphoma usually presents as unilateral or bilateral infiltration of diffuse large B‐cell lymphomas. We report a case of a 79‐year‐old man with near‐complete obstruction of the upper airways due to T‐cell prolymphocytic leukemia cells. Surgical resection was safely performed to reduce burden of disease.

## Graphical abstract

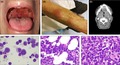

A 79‐year‐old man was referred to our hospital because of persistent sore throat, dysphagia, and shortness of breath over the past two months. Physical examination of the oral cavity revealed bilateral tonsillar hypertrophy resulting in the near‐complete obstruction of the upper airway (Figure 1A) and disseminated skin nodules (Figure 1B). A total‐body computed tomography confirmed tonsillar enlargement (Figure 1C), and widespread lymphadenopathy both above and below the diaphragm. Cell blood count showed leukocytosis WBC 44.000/μL, and flow cytometric analysis of bone marrow cells (Figure 1D) identified an abnormal T‐cell population expressing TCR α/β, CD2, CD3, CD4, CD5, CD7, and CD52. Cytogenetics confirmed a complex karyotype including an inversion of chromosome 14. These findings were indicative of T‐cell prolymphocytic leukemia (T‐PLL). Bone marrow biopsy (Figure 1E) and histological analysis of tonsillectomy specimen (Figure 1F) confirmed the T‐PLL lymphoid infiltrate.

**Figure 1:**
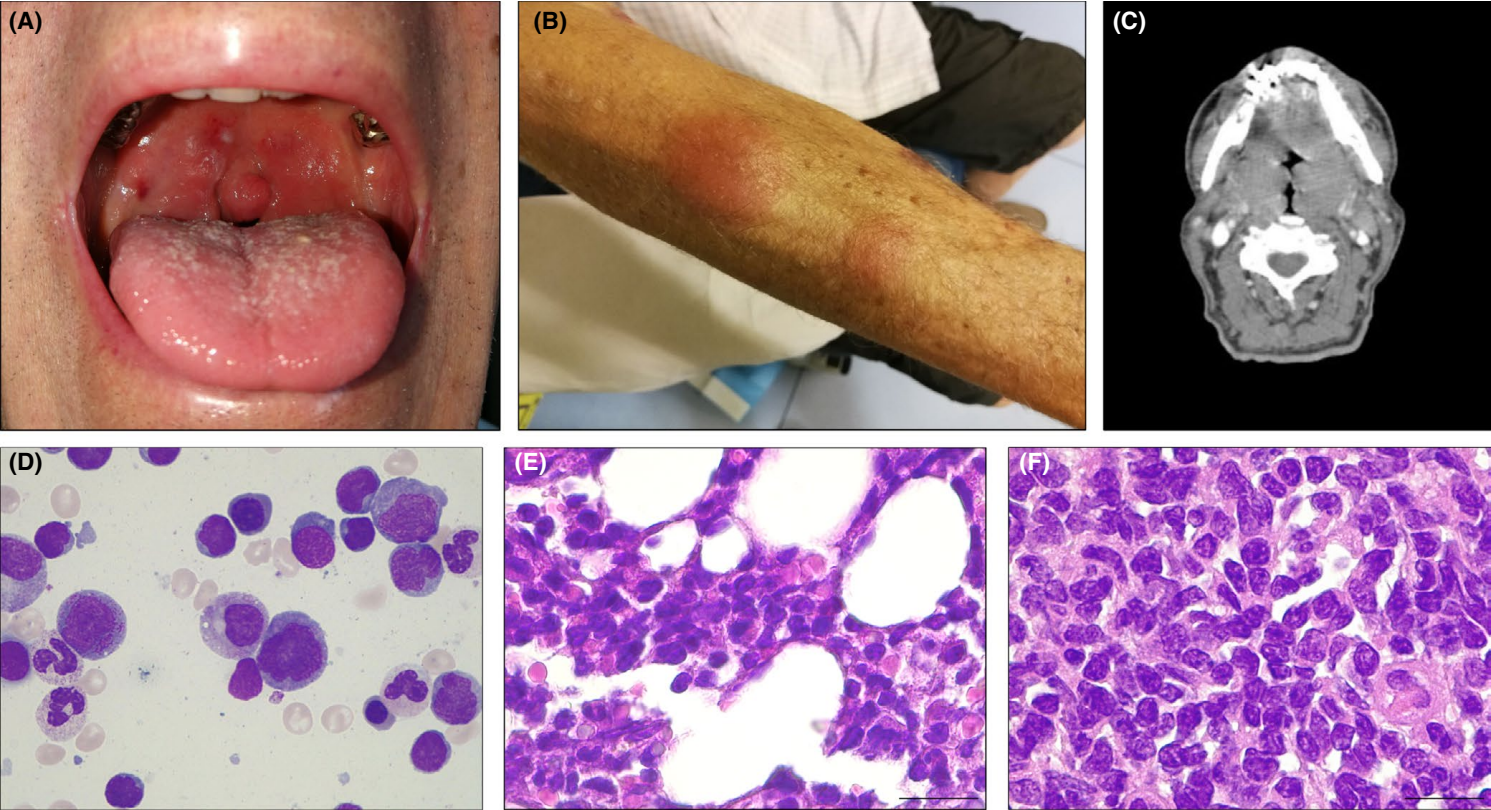
A, Bilateral tonsillar enlargement secondary to T‐cell prolymphocytic leukemia cells (T‐PLL) infiltration. B, Nodular skin infiltration. C, Axial contrast‐enhanced CT scan shows bilateral palatine tonsils enlargement. D, Bone marrow (BM) aspirate morphology showing T‐PLL cells. E, BM biopsy shows an abnormal lymphocytic infiltrate. F, Epithelium of tonsil showing diffuse infiltration of T‐PLL

T‐PLL is an aggressive cancer characterized by the hyperproliferation of post‐thymic prolymphocytes. T‐PLL accounts for ~2% of mature lymphocytic leukemias in adults.^1^ The majority of patients present with nodal involvement (58%), and splenomegaly is among the most common physical finding (38%).^2^ Skin lesions, including nodules, maculopapular rashes, or more rarely erythroderma, are described in ~20% of patients.^2^ T‐PLL is usually resistant to conventional chemotherapy, and complete remissions are rare including the one achieved with alemtuzumab‐based regimens.^3^

Because of patients’ life‐threatening condition, age, and severe risk of alemtuzumab infusion‐associated reactions, we initially tried cytoreducing the disease with age‐adjusted chemotherapy based on cyclophosphamide, vincristine, and high‐dose prednisone followed by the administration of 400 mg venetoclax for a month.^4^

However, after an initial response, we observed a worsening of clinical conditions that prompted surgical excision that improved the burden of the oral symptoms. While T‐PLL cutaneous infiltration is frequently observed in this disease, this is the first report describing a bilateral tonsillar involvement.

## CONFLICT OF INTEREST

The authors declare that there is no conflict of interest regarding the publication of this article.

## AUTHOR CONTRIBUTIONS

All authors contributed to the care of this patient. SDS and FDL: completed the surgical procedure. All other authors critically revised the manuscript. IB and GR: were responsible for the preparation of the manuscript. IB: collected patient's consent.
